# Autophagy is affected in patients with hypokalemic periodic paralysis: an involvement in vacuolar myopathy?

**DOI:** 10.1186/s40478-021-01212-8

**Published:** 2021-06-13

**Authors:** Thomas O. Krag, Sonja Holm-Yildiz, Nanna Witting, John Vissing

**Affiliations:** Copenhagen Neuromuscular Center, section 8077, Copenhagen University Hospital: Rigshospitalet, University of Copenhagen, Blegdamsvej 9, 2100 Copenhagen, Denmark

**Keywords:** Hypokalemic periodic paralysis, CACNA1S, Vacuoles, Autophagy, TFEB

## Abstract

Hypokalemic periodic paralysis is an autosomal dominant, rare disorder caused by variants in the genes for voltage-gated calcium channel Ca_V_1.1 (*CACNA1S*) and Na_V_1.4 (*SCN4A*). Patients with hypokalemic periodic paralysis may suffer from periodic paralysis alone, periodic paralysis co-existing with permanent weakness or permanent weakness alone. Hypokalemic periodic paralysis has been known to be associated with vacuolar myopathy for decades, and that vacuoles are a universal feature regardless of phenotype. Hence, we wanted to investigate the nature and cause of the vacuoles. Fourteen patients with the p.R528H variation in the *CACNA1S* gene was included in the study. Histology, immunohistochemistry and transmission electron microscopy was used to assess general histopathology, ultrastructure and pattern of expression of proteins related to muscle fibres and autophagy. Western blotting and real-time PCR was used to determine the expression levels of proteins and mRNA of the proteins investigated in immunohistochemistry. Histology and transmission electron microscopy revealed heterogenous vacuoles containing glycogen, fibrils and autophagosomes. Immunohistochemistry demonstrated autophagosomes and endosomes arrested at the pre-lysosome fusion stage. Expression analysis showed a significant decrease in levels of proteins an mRNA involved in autophagy in patients, suggesting a systemic effect. However, activation level of the master regulator of autophagy gene transcription, TFEB, did not differ between patients and controls, suggesting competing control over autophagy gene transcription by nutritional status and calcium concentration, both controlling TFEB activity. The findings suggest that patients with hypokalemic periodic paralysis have disrupted autophagic processing that contribute to the vacuoles seen in these patients.

## Introduction

Hypokalemic periodic paralysis (hypoPP) is an autosomal dominant, rare disorder with a prevalence of 1:100,000 that is caused by variants in the gene coding for calcium channel Ca_V_1.1 (*CACNA1S* OMIM 170,400) or less frequently in the gene for sodium channel Na_V_1.4 (*SCN4A* OMIM 613,345). Patients with hypoPP experience episodes of paralysis typically either spontaneously or after exercise or a meal or drink rich in carbohydrates. Affection in patients with the common variation p.R528H in the *CACNA1S* gene range from permanent paralysis, to periodic paralysis with (mixed weakness) or without permanent weakness [1]. Subjects with the p.R528H variant in *CACNA1S* have also been found to be asymptomatic. Severely affected patients with hypoPP often demonstrate dystrophic histopathology with variable fibre size, fibre regeneration and fibrofatty replacement of fibres, reminiscent of a muscular dystrophy [2, 3]. HypoPP has been described as a vacuolar myopathy for more than 50 years, and through light and electron microscopy studies much has been learned about the nature of the vacuoles [4–6]. A large genotype–phenotype study, involving both hypoPP and hyperkalemic periodic paralysis, demonstrated that a majority of the patients had vacuoles in myofibers [2]. Vacuoles are not uncommon in muscle diseases and are generally found in disorders affecting metabolism such as the glycogen storage disorders or in myofibrillar myopathies, however, in patients with hypoPP little is known about the origin of the vacuoles, why they are present and what they contain. We have very recently found that in patients with the p.R528H variant in *CACNA1S*, vacuoles are consistently present in the muscles of all patients [3], irrespective of phenotype and age. A study of the histopathology of muscle from these patients demonstrated that whereas all investigated muscles had vacuoles containing glycogen, not all vacuoles in a muscle section contained glycogen. Based on these observations, we hypothesized that part of the endosomal/autophagy pathway is affected by the disease due to alterations in calcium and/or energy homeostasis that could be momentarily disturbed and affect multiple cellular processes dependent on a proper calcium and energy homeostasis. Calcium ions control various stages of the autophagy process and sudden sarcoplasmic release of calcium ions may impede autophagosome-lysosomal turnover [7]. The mutation affects Ca_V_1.1 function, causing an inward leaking current leading to an opening of the ryanodine receptor 1 (RYR1) at the sarcoplasmic reticulum and thus an increase in cytoplasmic calcium [8]. This change in calcium concentration may change calcium homeostasis affecting multiple calcium dependent cellular systems and signalling pathways. The vacuoles in hypoPP patients suggest that at least glycophagy is affected and possibly other parts of the autophagic breakdown pathway. Changes in cytoplasmic calcium may activate or inhibit autophagy, and changes in lysosomal and possibly also cytoplasmic calcium may affect translational control of the CLEAR (Coordinated Lysosomal Expression and Regulation) network of autophagy-related genes [9–12]. We hypothesize that this change in calcium homeostasis affects the autophagosomal and endosomal pathways in patients with hypoPP. Based on the vacuoles in muscle tissue we set out to demonstrate if the vacuoles contained organelles involved in the endosomal/autophagosomal pathway, and if so, to what extent this pathway was affected in the muscles of hypoPP patients and a possible cause for the vacuoles and affection of autophagy in the muscles by investigating the presence and expression of a selection of genes and proteins involved in autophagy.

## Patients and methods

### Patients

Fourteen patients with genetically verified hypoPP harbouring the p.R528H mutation in the *CACN1AS* gene were included in the study. Nine were affected by periodic paralysis (6 males/3 females; age 28 ± 10 years old) while 6 patients were affected by mixed weakness (3 males/3 females; age 63 ± 14 years old). Muscle biopsies were taken from the *vastus lateralis* using a Bergstrøm needle, a piece was flash frozen in isopentane cooled in liquid nitrogen and stored at − 80 °C and a piece was fixed in 2% glutaraldehyde for minimum 48 h in phosphate buffer at 4 °C. For western blotting and quantitative PCR, a subset of patients (3 males/4 females; age 38 ± 20 years old) were studied. The study included 5 healthy controls (1 males/4 females; age 56 ± 15 years old).

### Histology and immunohistology

In order to assess the general histopathology and more specifically, the content of vacuoles a series of histological stains were made, in addition to immunohistology for a range of proteins involved in sarcomeric structure and autophagy. Biopsies were sectioned on a cryostat in 10 µm slices and stained with haematoxylin & eosin for general histopathological evaluation, with periodic Schiffs reagent (PAS) for glycogen and for acid phosphatase using a naphtol AS-BI based standard protocol. For immunohistology, sections were fixed in 10% neutral-buffered formaldehyde or ice cold acetone, blocked in 3% foetal calf serum in phosphate-buffered saline (PBS) and incubated over night at 4 °C with antibodies at a concentration of 1:100, unless otherwise stated, against myofibrillar proteins and proteins involved in the endosomal/autophagy pathway using antibodies against acid α-glucosidase (GAA, ab102815), desmin (ab32362; 1:200), early endosome antigen 1 (EEA1, ab2900), rab5 (ab18211), rab7 (ab50533), rab11 (ab3612), microtubule-associated light chain 3 (LC3B, ab192890), atg5 (ab108327), beclin-1 (ab62557), lysosome-associated membrane glycoprotein 2 (LAMP2, ab13524), p62/SQSTM1 (ab56416) all from Abcam (Cambridge, UK), skeletal muscle actin (VP-M659, Vectorlabs, Burlingame, CA; 1:200), filamin C (HPA006135, Sigma-Aldrich, St. Louis, MO). Antibody against C5b-9 (M0777, Agilent Technologies, Glostrup, Denmark) was used to visualize membrane attack complex (MAC) deposits. All sections were incubated with antibodies against laminins (L9393, Sigma-Aldrich; 1:500) or dystrophin-2 (DYS2, Leica Biosystems, Wetzlar, Germany) in addition to the above antibodies in order to stain the sarcolemma or extra-cellular matrix. After washing, sections were incubated with Alexa Fluor 488 and 594 goat anti-rabbit and goat anti-mouse (Thermo Fisher Scientific, Waltham, MA) for an hour at room temperature followed by a brief incubation with the nuclear stain 4’,6-diamidino-2-phenylindole (DAPI, Thermo Fisher Scientific). Laminin and dystrophin were always stained with Alexa Fluor 488. Additional sections were stained for LAMP2 (Clone H4B4, DSHB) and developed using 3,3′-Diaminobenzidine (DAB) according to manufacturer’s instructions (Agilent, Glostrup, Denmark) to demonstrate proper sarcoplasmic expression outside of strongly stained vacuoles as seen in AlexaFluor stained sections. All sections were investigated in a Nikon Ti-E microscope at 20 × or 40 × and images were taken with a Nikon DS-5Mc, DS-Fi3 or an Andor Neo camera using NIS Elements software (Nikon, Tokyo, Japan). Pictures in different colour channels were merged using the function in software.

### Transmission electron microscopy

To visualize the ultra-structure and the vacuoles in the muscle biopsies, we performed transmission electron microscopy (TEM) on patient and mouse samples using a protocol previously described [13]. Briefly, a piece of fresh muscle biopsy was perfused with 2% electron microscopy grade glutaraldehyde in 0.05 M phosphate buffer. After post-fixation and staining, the biopsy was embedded in Epon and sectioned both at a transversely and longitudinal orientation. Sections were visualized in a CM100 transmission electron microscope (Philips, Amsterdam, Netherlands) fitted with a 4Mpixel Veleta camera (Olympus Soft Imaging Solutions GmbH, Münster, Germany). EM images were acquired with a specific focus on glycogen pools and vacuoles.

### Western blotting

In order to determine how protein expression related to the immunohistology, we did western blotting for most of the proteins related to autophagy that were immunostained for. Western blotting was carried out as previously described [13]. Briefly, muscle biopsies were cut on a cryostat and sections were homogenized in lysis buffer, separated on sodium dodecyl sulfate–polyacrylamide gel (SDS-PAGE) gel and blotted onto polyvinyl difluoride (PVDF) membrane. Membranes were incubated in primary antibodies over night at 4°. The following antibodies were used at 1:1000: atg5, beclin-1, GAA, LAMP1, LAMP2, LC3, p62/SQSTM1, rab5 and rab7 (Abcam), transcription factor EB (TFEB), TFEB phospho-Ser211 (#4240 and #37,681, Cell Signaling Technologies, Danvers, MA) and TFEB phospho-Ser142 (ABE1974-I, Sigma-Aldrich). Membranes were subsequently incubated with a secondary antibody goat anti-rabbit/mouse-HRP at 1:10,000 (Agilent, Glostrup, Denmark) for 3 h at room temperature and developed using Clarity Max (Bio-Rad, Hercules, CA) and visualized in a ChemiDoc MP digital darkroom (Bio-Rad).

### Quantitative PCR

In order to assess if changes in protein expression between patients and controls were related protein degradation or changes at the transcriptional level, we performed qPCR on a subset of genes involved in autophagy that we had studied using western blotting. RNA samples were obtained from the muscle biopsies, following the instructions of Trizol (Thermo Fisher Scientific). cDNA was synthesized from 750 ng of muscle total RNA using the iScriptTM cDNA Synthesis Kit (Bio-Rad). qPCR was performed using a CFX96 Real-Time PCR System (Bio-Rad), with the following TaqMan fluorogenic probes (Thermo Fisher Scientific):

(i) rab5A gene (RAB5A, Hs00702360_s1); (ii) rab7A gene (RAB7A, Hs01115139_m1); (iii) p62/SQSTM1 gene (SQSTM1, Hs01061917_g1); (iv) LC3 gene (MAP1LC3A, Hs01076567_g1); (v) beclin-1 gene (BECN1, Hs01007018_m1); (vi) Atg5 gene (ATG5, Hs00169468_m1) and (vii) LAMP1 gene (LAMP1, Hs00931461_m1). Results were normalized to peptidylprolyl isomerase A mRNA levels (PPIA, Mm02342430_g1).

## Results

### Morphology and transmission electron microscopy ultrastructural analysis

**Fig. 1:**
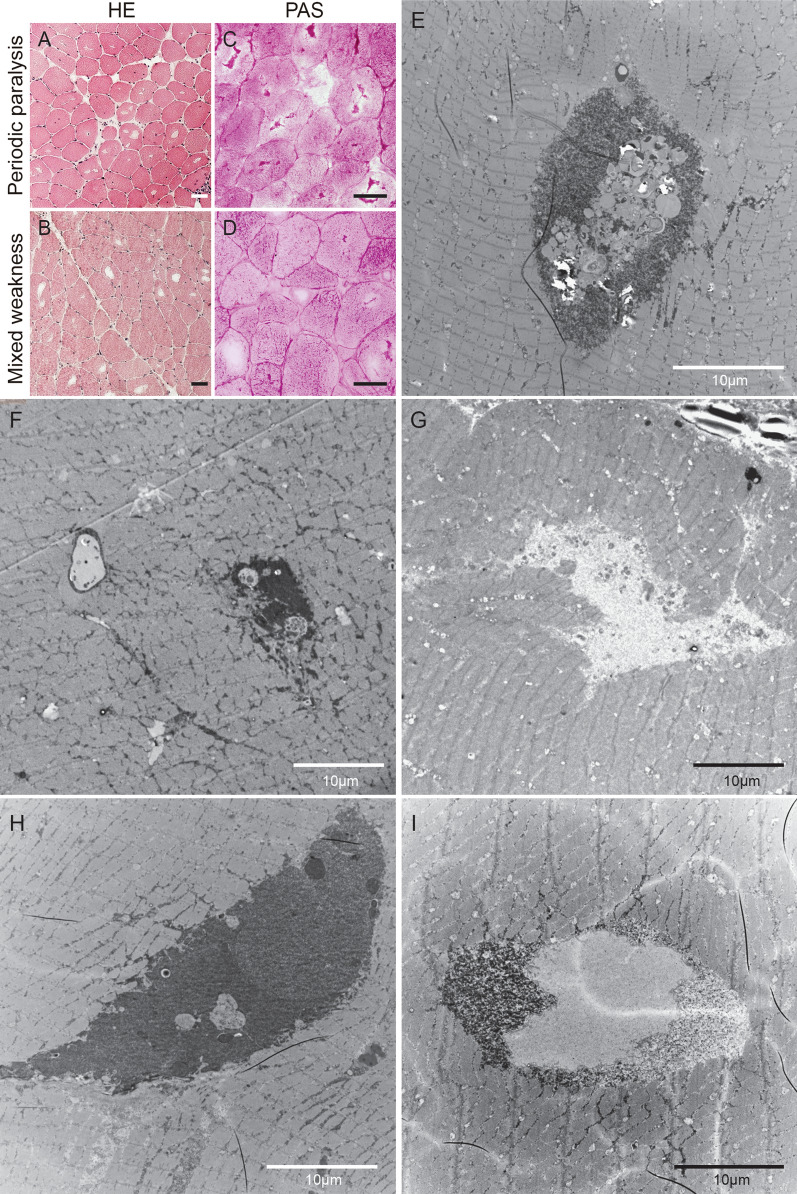
The heterogenous content of vacuoles in muscles of patients with hypokalemic periodic paralysis. **a,b.** Haematoxylin & Eosin stained sections from a patient with periodic paralysis (**a**) and mixed weakness (**b**) demonstrate vacuoles in many muscle fibres. **c,d.** Periodic acid Schiffs stain demonstrate that many but not all of the vacuoles in muscles from a patient with periodic paralysis (**c**) and mixed weakness (**d**) contain glycogen. **e-i.** Low magnification transmission electron microscopy (EM) reveal that the vacuoles may have a very heterogenous content, embedded autophagosomes surrounded by glycogen (**e**), very large autophagosome (**f**), fibrillar content (**g**), large accumulation of free glycogen (**h**) and accumulations of glycogen combined with fibrillar content and membrane-encapsulated glycogen (**i**). Bar in histology images is 50 µm and in EM images 10 µm

All patients with hypoPP demonstrated vacuoles, regardless if the patients had periodic paralysis or a mix between periodic paralysis and permanent weakness (Fig. 1a, b). Most, but not all vacuoles, contained glycogen regardless of size of the vacuoles (Fig. 1c, d). A collapse of the sarcotubular system was also evident in some vacuoles (Fig. 1d). Low power transmission electron microscopy (TEM) demonstrated that the vacuoles indeed contained glycogen and autophagosomes, often in the same vacuoles, but very large autophagosomes could also be found separately (Fig. 1e, f). Some vacuoles did not contain any glycogen but organelles in various stages of the autophagy pathway as well as protein fragments, whereas other vacuoles mostly contained glycogen (Fig. 1h, i). The difference in glycogen granule density was substantial (Fig. 1i).

The vacuoles evidently change the ultrastructure of the myofiber, bringing Z-lines out of register, without causing Z-line disruption or instability. This broke up microfibrils as the vacuoles occupied an amorphous three-dimensional space (Fig. 1i).

**Fig. 2:**
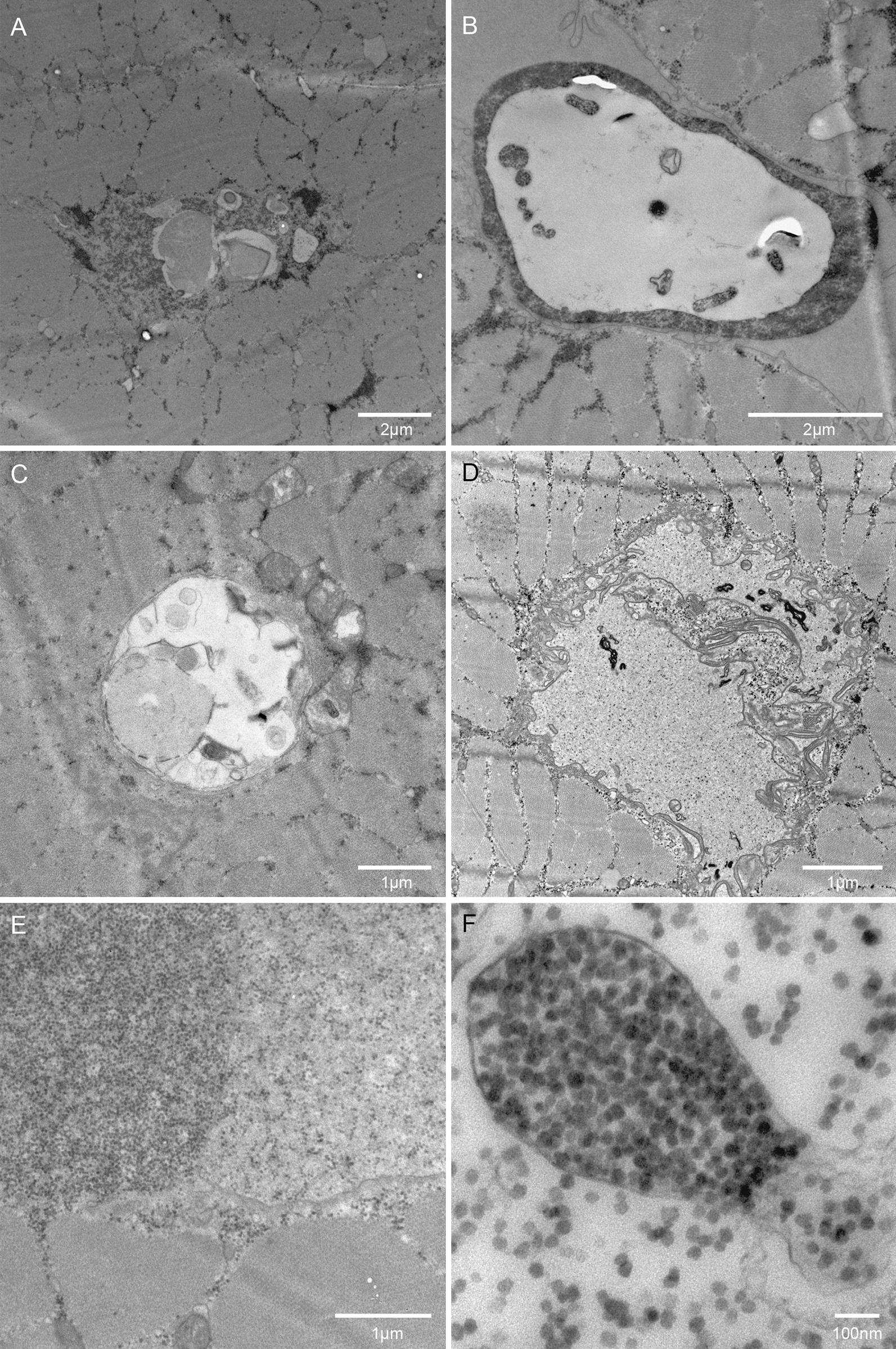
Ultrastructure appears affected by organelles arrested in the autophagic processing. **a-c.** High magnification electron microscopy images demonstrate various stages of autophagosome formation, type and size. **d.** Autophagic degradation of endoplasmic reticulum. **e.** The coexistence of free glycogen and membrane-encapsulated glycogen, possibly very large lysosome. **f.** Very high magnification of lysosome containing glycogen

At higher magnification, large to very large endosomes/autophagosomes (2–6 µm across) were seen, again at different stages of the breakdown cycle (Fig. 2a–c). Of particular interest was an organelle with breakdown products inside other than glycogen, surrounded by a layer of glycogen, which unlike most other pools of glycogen was surrounded by membranes on either side of the layer (Fig. 2b). Vacuoles did also contain protein fragments and layered membranes (Fig. 2d). Vacuoles containing glycogen sometimes had separate high and low density of glycogen granules separated by a membrane or vesicles with a high density of glycogen granules situated in a larger vacuole with a low density of glycogen granules (Fig. 2e, f).

**Fig. 3:**
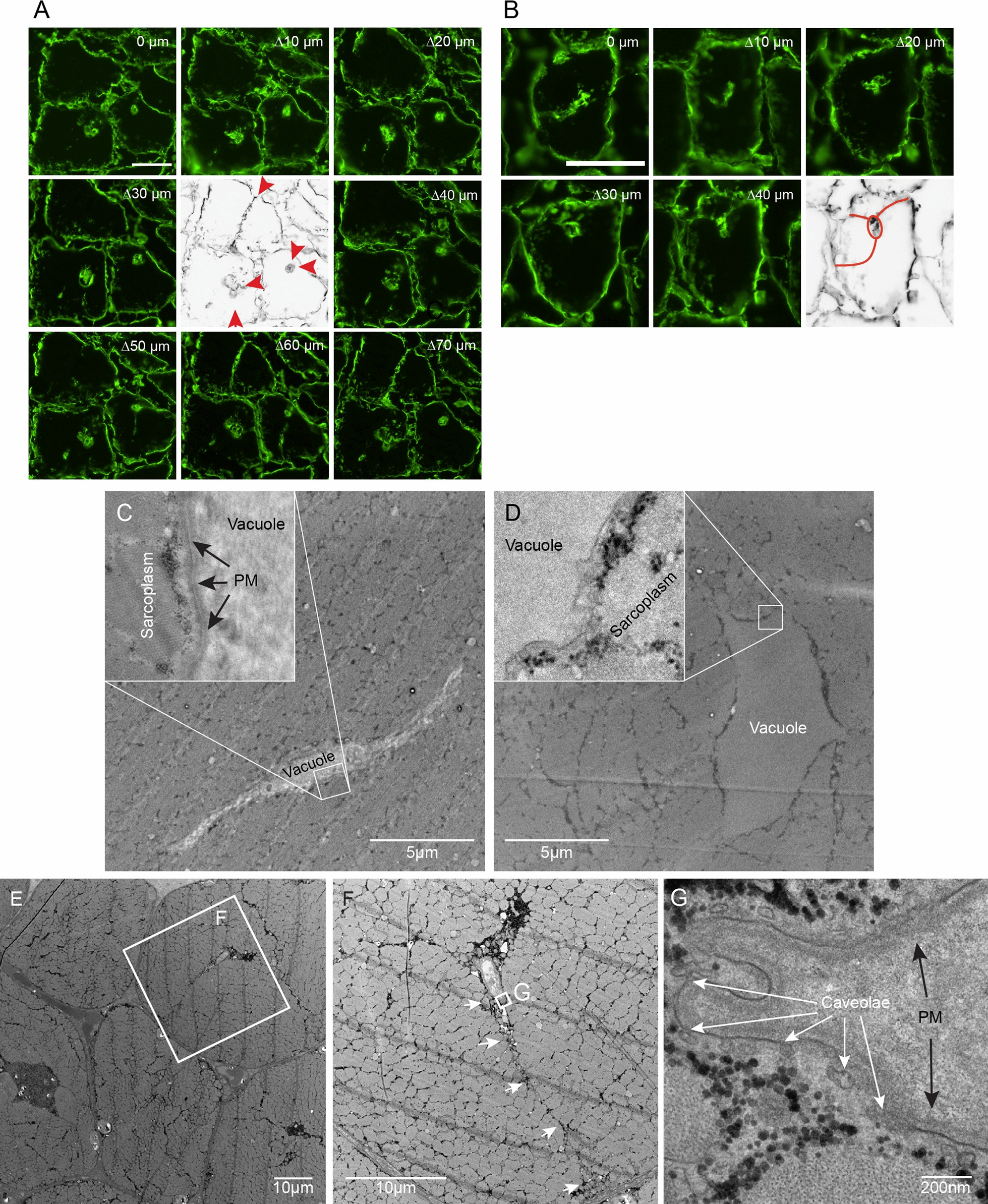
Vacuoles may originate from membrane invaginations. **a.** Serial sections stained for laminin with a section thickness of 10 µm demonstrate how invaginations apparently terminate in three vacuoles (red arrowheads), bar is 50 µm. **b.** Serial sections demonstrate how invaginations (red line) from opposite sides meet at a vacuole (red oval), bar is 50 µm. **c,d.** electron microscopy images demonstrating vacuoles with no content and plasma membrane (PM, black arrows) separating sarcoplasm from vacuole (inserts). **e,f.** Low power electron microscopy image demonstrating different vacuoles, one of which is connected to the outside of the myofiber by an invagination (white arrows). The white box in panel E refers to panel F, and the box in panel F refers to panel G. **g**. An invagination ends in “fingers” with caveolae (white arrows) along the plasma membrane (black arrows)

An analysis of all patients in terms of ultra-structural changes based on TEM revealed that the majority of patient biopsies had free interfibrillar and subsarcolemmal glycogen, vacuoles with glycogen, large autophagosomes, vacuoles with fibrillar protein and central nuclei, a feature of myofiber regeneration (Table 1). There was no correlation between age, sex, phenotype and the ultra-structural findings. Interestingly, myofiber invaginations were found in all biopsies. Serial sections of muscle stained for laminin reveal that invaginations may terminate in a vacuole (Fig. 3a). In a similar fashion, multiple invaginations may terminate in a vacuole, or form a vacuole and proceed to another exit point at the myofiber surface (Fig. 3b). TEM images reveal empty vacuoles with plasma membrane suggesting they are part of an invagination (Fig. 3c, d). However, invaginations are clearly also ending in vacuoles with glycogen and autophagosomal content, even though they may appear as remnants where part of the invagination could be fused (Fig. 3e, f). Enlarging a vacuole reveal caveolae along the plasma membrane, which is part of normal endocytosis, as well as “fingers” extending from the plasma membrane in an invagination (Fig. 3g).

**Table 1:** Phenotype, demographics and ultrastructure analysis of patients with hypokalemic periodic paralysis

ID	Phenotype PP/MW	Sex M/F	Age years	Free Glygogen	Glycogen vacuole	Large Autophagosomes	Fibrillar vacuole	Central nuclei	Sarcolemmal invagination
1	PP	M	20	Yes	Yes	Yes	No	Yes	Yes
2	MW	F	46	Yes	Yes	Yes	No	Yes	Yes
3	PP	M	49	No	Yes	Yes	No	Yes	Yes
4	MW	F	73	Yes	Yes	Yes	Yes	Yes	Yes
5	PP	F	30	No	Yes	Yes	No	Yes	Yes
6	MW	M	52	Yes	Yes	Yes	Yes	Yes	Yes
7	PP	M	21	Yes	Yes	Yes	Yes	Yes	Yes
8	PP	F	30	Yes	Yes	Yes	Yes	Yes	Yes
9	PP	M	21	No	Yes	Yes	Yes	Yes	Yes
10	MW	M	68	Yes	Yes	Yes	Yes	Yes	Yes
11	PP	M	18	Yes	Yes	no	Yes	Yes	Yes
12	PP	M	24	Yes	Yes	Yes	Yes	Yes	Yes
13	PP	F	26	Yes	Yes	Yes	Yes	Yes	Yes
14	MW	F	52	Yes	Yes	Yes	Yes	Yes	Yes
total	9/5	8/6	38 ± 19	11/14	14/14	13/14	10/14	14/14	14/14

W: mixed weakness; PP: periodic paralysis; M: male; F: female; Age: age when biopsy was taken

### Protein content and deposits in the vacuoles

**Fig. 4:**
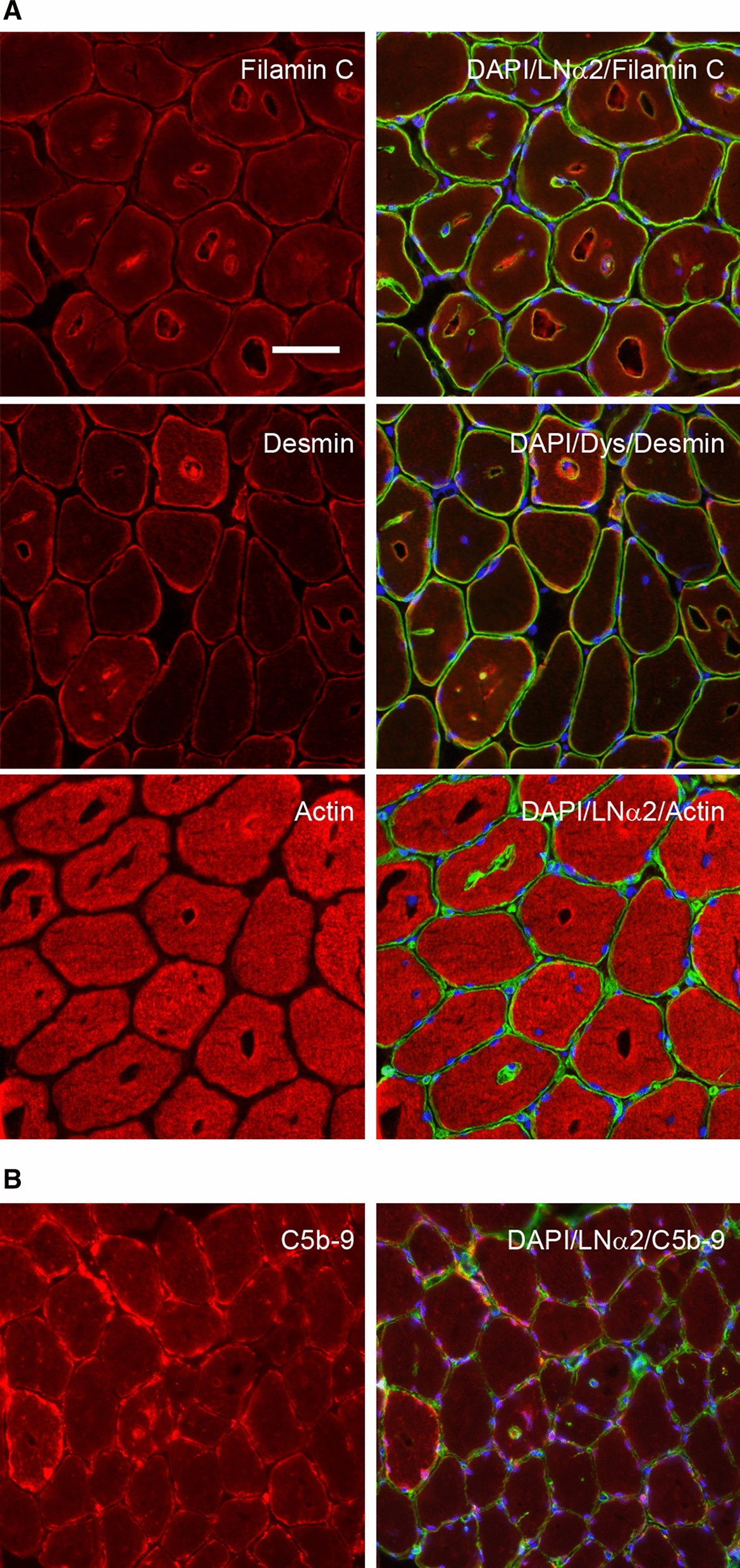
Filament but not sarcomeric proteins in vacuoles and membrane attack complex may line the membrane. **a.** Fluorescence microscopy reveal that filamin C protein (FLNC) and desmin may accumulate in the vacuoles whereas the sarcomeric protein actin does not. Both laminin α2 (merosin) and dystrophin (LNα2 & DYS in green), used to demonstrate the sarcolemma clearly line the vacuoles as well and in the case of laminin α2 this, appear to accumulate in the vacuoles as well. DAPI (blue) demonstrates nuclei and the merged pictures show that sporadic vacuoles may contain nuclei. Several fibres demonstrate invaginations in the laminin α2 and dystrophin stains. **b.** Membrane attack complex complement C5b-9 deposits are seen in fibres with vacuoles, both at the sarcolemma, membrane invaginations and inside vacuoles. Bar is 50 µm

In order to determine if the vacuoles contained protein, we stained for filament and sarcomeric protein, but whereas vacuoles were filamin C and desmin positive, both at the lining and the inside of the vacuoles and not necessarily at the same time, they were actin negative (Fig. 4a) consistent with our diagnostic stains for myosin heavy chain type I/II, which demonstrated equal distribution of vacuoles in both fibre types, but no myosin in vacuoles, and thus no sarcomeric protein in the vacuoles (not shown).

A stain for C5b-9 demonstrated deposits at the plasma membrane surface, abnormal membrane invagination as well as vacuolar lining deposits, the latter likely from endocytosed MAC (Fig. 4b). In the course of the immunohistochemical analysis, the presence of sarcolemmal invaginations were clearly visible in stains for dystrophin and laminin, consistent with TEM findings.

**Fig. 5:**
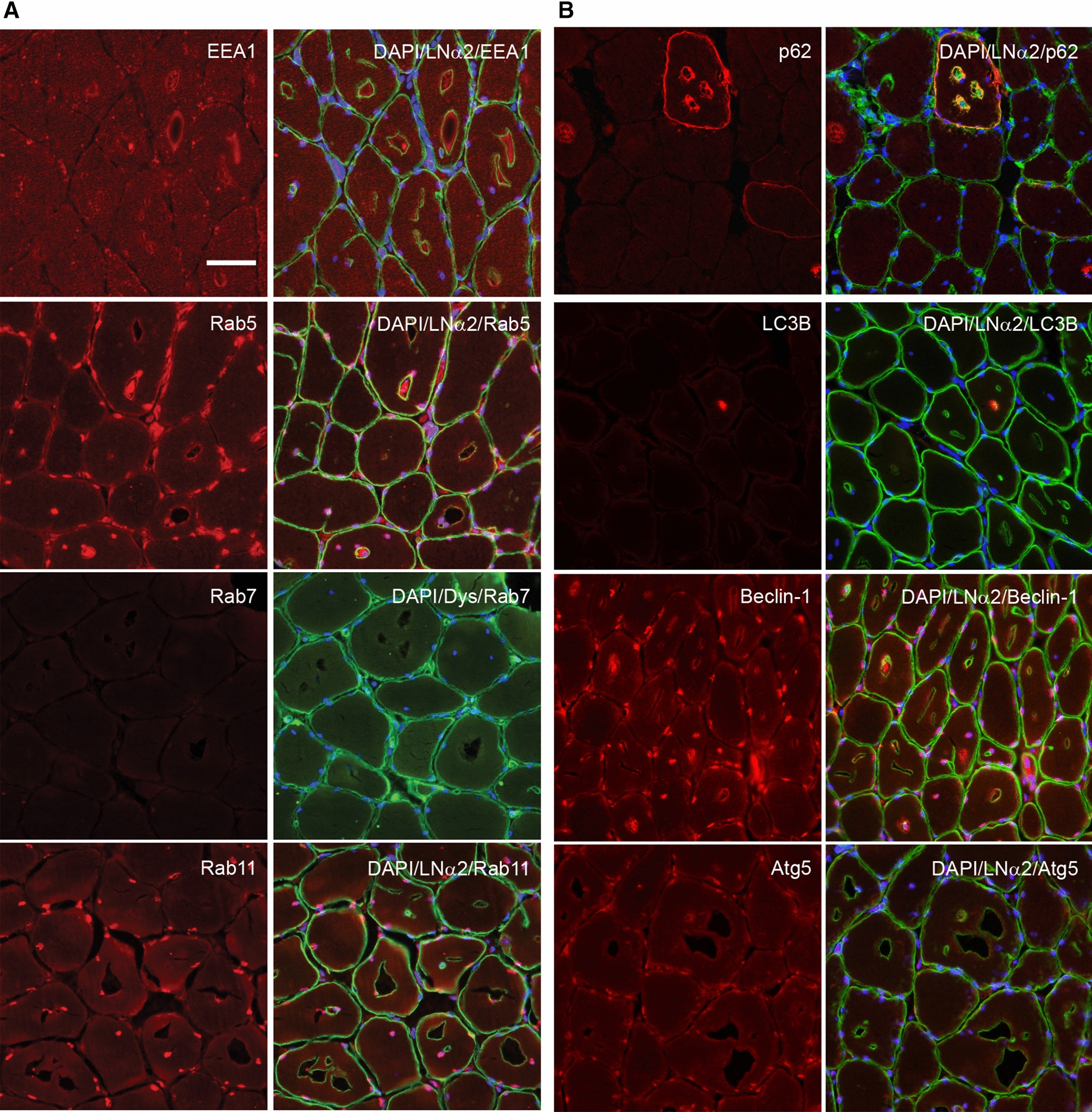
The endosome pathway appears affected in muscle from patients with hypokalemic periodic paralysis. **a.** The early endosome markers EEA1 and rab5 appear to accumulate in vacuoles, while the late endosome marker rab7 and endosome recycling marker rab11 does not, suggesting intermittent endosome processing arrests. **b.** The autophagosomal pathway appears affected in muscle from patients with hypokalemic periodic paralysis. The autophagophore markers p62/SQSTM1 and beclin-1 clearly accumulate in some vacuoles while LC3 often appears dissociated from p62 and Atg5, a late autophagosomal marker does not accumulate in the vacuoles at all. Like for endosome processing, the autophagosomal marker stains suggest an intermittent processing arrest between early and late stage. Bar is 50 µm

The presence of large autophagosomes/endosomes in TEM-pictures prompted stains for early, late and recycling markers for endosomes, EEA1 and rab5/7/11. Only rab7 was not seen in the vacuoles suggesting that the vacuoles contained early endosomes and recycling endosomes but not late endosomes visible in fluorescent microscopy. Specifically, EEA1 and rab5 and 11 stained the inside, but not the lining of the vacuoles (Fig. 5a).

Similar to staining for endosomes, we also stained for different stages of the autophagosomal pathway, the autophagophore, early and late autophagosomes. LC3, a marker for autophagophore, was only found sporadically inside vacuoles, while p62/SQSTM1 was found lining vacuoles and beclin-1 was found inside, but not lining a substantial number of vacuoles and atg5 was not detected in any vacuole (Fig. 5b). These results suggest that early stage autophagosomes are predominantly found in the vacuoles and an occasional arrest in endosome/autophagosome maturation from early to late stage.

**Fig. 6:**
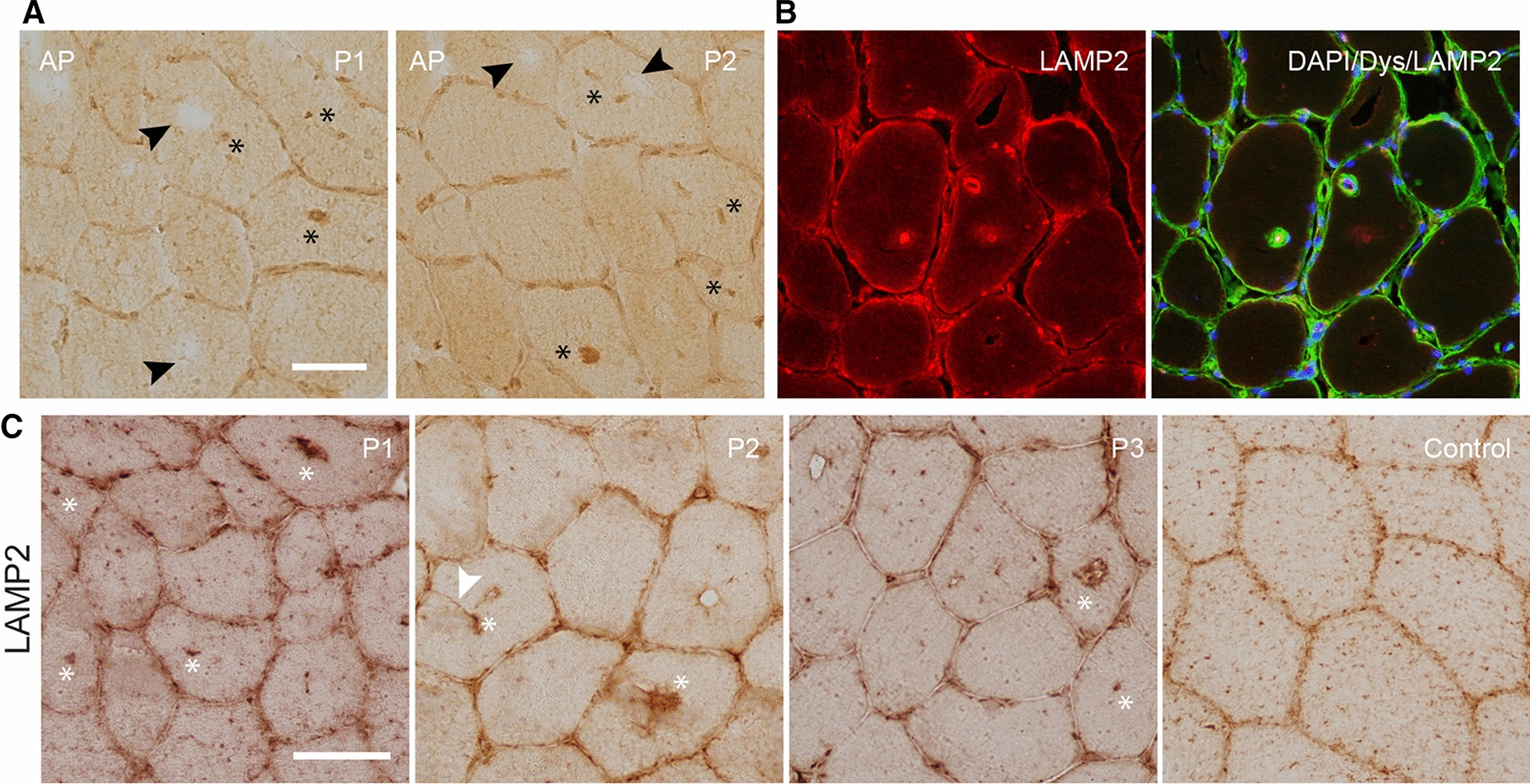
Vacuoles may contain very large lysosomes. **a.** Acid phosphatase-stained muscle sections from two representative patients demonstrates positive vacuoles (asterisk) suggestive of large lysosomes, while other vacuoles remain acid phosphatase negative (black arrowhead). **b.** The lysosomal marker LAMP2 can been seen prominently in some vacuoles consistent with the acid phosphatase positive vacuoles. Laminin α2 (merosin) and dystrophin (LNα2 & DYS in green) were used to demonstrate the sarcolemma while DAPI (blue) was used to demonstrate nuclei. c. Sections from three representative patients (P1-3) and a control demonstrate very large LAMP2-positive vacuoles (white asterisk), which are absent in the control. In addition, an invagination leading to a LAMP2-positive vacuole is evident in patient 2 (white arrowhead). Bar is 50 µm

Staining for acid phosphatase demonstrated that some vacuoles were positive for acid phosphatase suggestive of lysosomes (Fig. 6a). Staining for LAMP2, a lysosomal marker, demonstrated that some, but not all, vacuoles contained lysosomes consistent with the single membrane organelles containing glycogen found in TEM-images as well as the acid phosphatase positive vacuoles (Fig. 6b). In patients, LAMP2 appears to be expressed in fewer spots and more concentrated in the vacuoles compared to the uniform spread of LAMP2-positive lysosomes in normal myofibers (Control) (Fig. 6c). As opposed to the endosomal and autophagosomal proteins, LAMP2 was found both inside and lining the vacuoles.

### Autophagy protein and mRNA expression and transcriptional control of autophagy gene expression

**Fig. 7:**
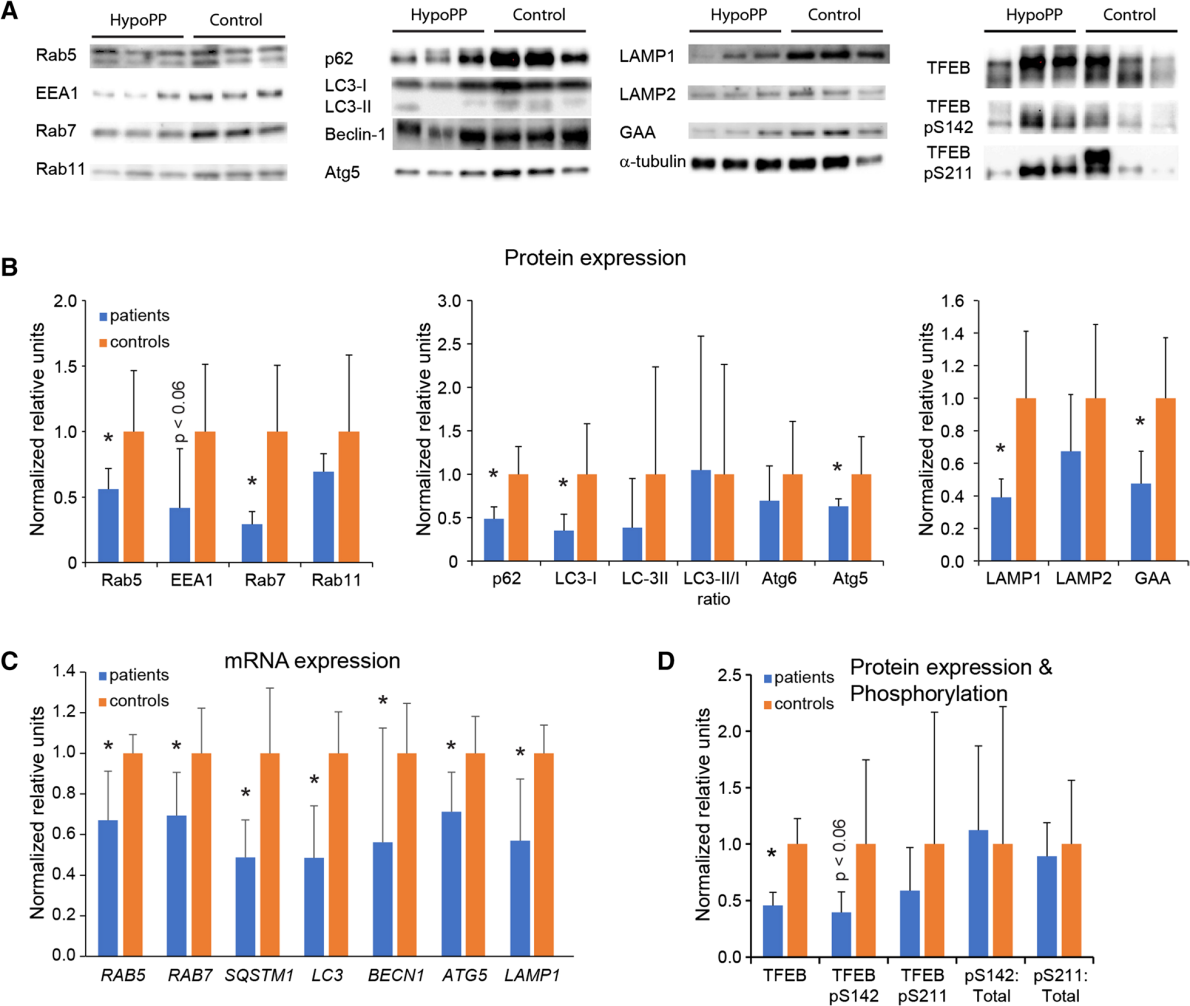
Expression of mRNA and proteins involved in autophagy is decreased. **a.** Representative western blots of the proteins investigated in **b** and **d** for three patients with hypoPP and normal controls (Control). **b.** Several proteins involved in autophagy were decreased in hypoPP patients suggesting a systemic suppression of expression in the hypoPP patients and EEA1, an early endosomal marker demonstrated a trend toward a decrease (*p* < 0.06). **c.** mRNA analysis demonstrated that expression of selected autophagy genes was decreased. **d.** Protein expression of the master regulator of autophagy gene expression, TFEB, and level of phosphorylation at the nuclear translocation related serine 142 and 211 demonstrates that there is no difference between patients and controls in terms of ratio of phosphorylated serines to total level of TFEB

In addition to the abnormal TEM and immunohistology findings, we also wanted to determine if the expression of proteins involved in the autophagy pathway was changed. In the endosome, we found that expression of rab5 and rab7 was significantly reduced, whereas EEA1 demonstrated a trend of reduction (*p* < 0.06) (Fig. 7a). No difference was found for rab11. In the autophagosomal pathway, we found that p62/SQSTM1, LC3-I and atg5 were significantly reduced, while the LC3-II/I ratio was the same as for controls. Among markers for lysosomes, LAMP1 and GAA were significantly reduced.

Subsequently we performed quantitative polymerase chain reaction (qPCR) on a subset of the mRNA’s we had studied protein expression onto determine if the decreased level of protein was caused by a low gene expression level and found that all genes investigated (*RAB5A, RAB7A, SQSTM1, MAP1LC3A, BECN1, ATG5* and *LAMP1*) had decreased mRNA expression in patients compared to controls (Fig. 7b).

The decreased level of mRNA expression led to investigation of the activity of transcription factor EB (TFEB), a master regulator of many autophagy genes, and dephosphorylation of serine 142 and 211 leads TFEB to translocate to the nucleus. While the level of TFEB was significantly reduced in the patients, the phosphorylation level was trending lower for TFEB phosphoserine 142 (*p* < 0.06). The ratio of TFEB phosphorylated serine 142 and 211 to total TFEB was the same for patients and controls suggesting no difference in activity of translocating to the nucleus (Fig. 7c).

## Discussion

The main findings of this study are that all investigated patients have heterogenous vacuoles in size and content that to some extent related to disturbances in the autophagic processing of cellular organelles, proteins and glycogen. Invaginations from the sarcolemma may connect to or terminate at vacuoles. We also find that autophagy in these patients is affected as autophagosomes/endosomes are found arrested to some extent in the early to late transition and that expression of key proteins involved in the autophagosomal/lysosomal pathway is significantly reduced. Consistent with this, this study also finds that all investigated autophagy genes had decreased expression. The presence of terminal MAC deposits at the plasma membrane suggests a more complex interaction between MAC, autophagic processing and calcium concentration leading to collateral damage as sarcolemmal invagination suggests loss of proper membrane repair and/or increased calcium mediated endocytic activity.

While it has been known for more than 50 years that hypokalemic periodic paralysis was also a vacuolar myopathy [5], the cause for this has been unknown. The heterogenous appearance of free and membrane-bound glycogen as well as fibrillar content and various stages of autophagosomes in TEM images, suggests that a broader spectrum of autophagy is affected. The fact that only fibrillar protein and no sarcomeric protein is found in the vacuoles, also suggests that the integrity of the myofibril bundling is affected, leading to local but not systemic loss of Z-line register and random breaks in the sarcomeric structure. This is in contrast to myofibrillar myopathies with autophagic vacuoles. In addition, we found collapses of the sarcotubular system in some of the vacuoles, suggesting that less dense organelles become collateral damage, once vacuoles form and become filled with cellular debris, glycogen, autophagosomes expand outwards and crush anything less dense than sarcomers, as we have previously found in the mouse model of McArdle disease [13]. This may long-term affect muscle function and explain the permanent weakness in some of the hypoPP patients [1]. For this reason, we investigated the presence of endosomal, autophagosomal and lysosomal proteins in the vacuoles using selected markers for various stages of autophagic processing. The fact that the vacuoles did not appear to be fibre type specific suggests that a mechanism independent of fibre type cause the disturbances in autophagy and thus the vacuoles [1]. This immunohistochemical investigation led to a mapping of what appeared to be intermittent autophagic processing issues that is focused between early and late transition of endosomes and autophagosomes in a process that is calcium-dependent [14]. The concomitant affection of both the autophagosomal and endosomal processing pathway at the transition between early and late stage suggests a systemic effect on the transition, part of which has previously been demonstrated in Lafora disease where both autophagosomal and endosomal proteins were found in vacuoles [15]. Interestingly, the immunohistochemical stain for p62 and LC3 demonstrated that p62 localization often is dissociated from LC3, in particular when localized strongly at the sarcolemma. This suggested an LC3-independent p62-targeting to the autophagosome [16]. Additionally, the finding of acid phosphatase and LAMP2 positive vacuoles is consistent with the finding of membrane-enclosed glycogen pools in electron micrographs, presumably large lysosomes unable to fuse with autophagosomes. It can be speculated that this is caused by the intermittent stoppage in early to late transition of autophagosomes, where early large autophagosomes are unable to properly fuse with lysosomes. When comparing the distribution of LAMP2 positive stain between patients and controls, it is clear that patients generally have far fewer, but much larger LAMP2 positive lysosomes compared to controls, consistent with the LAMP protein and mRNA expression results in this study.

Disturbed autophagy has been found in several different groups of muscle diseases, excellently reviewed by Castets et al. [17]. Among these, a particular group of diseases, autophagic vacuolar myopathy, to which Danon’s disease (LAMP2-deficiency) and X-linked myopathy with excessive autophagy (XMEA) belong prominently, feature large autophagosomes in vacuoles similar to our observations [18–22]. Also, Pompe disease (glycogen storage disease type II) is considered an autophagic vacuolar myopathy [23, 24]. The common mechanism is a block at a late step in the autophagic pathway caused by an increase in proteins but not in mRNAs involved in autophagy as well as an increased LC3-II/I ratio. The decrease in mRNA and protein expression in hypoPP patients makes it clear that hypoPP is not an autophagic vacuolar myopathy despite some histological similarities. Autophagic processing may relate to changes in gene expression of autophagy-related genes, which a number of studies have demonstrated by showing upregulation of TFEB, a master activator of a large number of autophagy-related genes [25]. However, both are dependent on and respond to changes in calcium concentration for proper function. Evidence suggests that the relation between calcium and autophagy is a double-edged sword where changes in calcium concentration may lead to activation or inhibition of autophagy [26]. Generally, an increase in cytosolic calcium leads to increased autophagy beyond a basic level. In the hypoPP patients, there appear to be two contributing factors to an increase in calcium, one being an increased opening of ryanodine receptor 1 in sarcoplasmic- and endoplasmic reticulum due to the leaking Ca_V_1.1, the other, potentially much more serious, is the MAC formed pores in the membrane, which let in extra-cellular calcium. The presence of MAC deposits has been demonstrated in multiple myopathies, generally at fibres entering the necrotic process, but of particular interest to this study also in XMEA [27, 28]. However, the MAC deposits have also been found on the surface of non-necrotic fibres at levels that are sublytic through a yet unknown mechanism [29]. The increase in calcium due to MAC deposits may lead to abnormal autophagy, an increase in endocytosis as well as lysosomal exocytosis [30, 31]. MAC has previously been demonstrated to cause blockage of the autophagic pathway at the autophagosome-autolysosome transition leading to accumulation of autophagosomes [32]. This could present one explanation for the presence of accumulated autophagosomes in the vacuoles. Whereas the complement system has been known to activate autophagy as a response to unwanted cellular waste or pathogens, the mechanism that triggers a complement response and MAC deposits at the muscle fibres can be caused by multiple factors [33].

The presence of sarcolemmal invaginations in various myopathies is scarcely reported in the literature as an independent finding and a poorly understood phenomenon [20, 28, 34, 35]. It is possible that caveolae, part of normal endocytosis, may not close and release from the plasma membrane under certain circumstances. As the internalization of caveolae to endosomes may be a rab5-dependent pathway [36] and rab5 expression is decreased in hypoPP patients, the extended caveolae may turn into extended invaginations before internalization takes place. Two things may happen next, one is internalization into an endosome, the other is a fusion of the two opposing membranes in an invagination in a calcium-dependent manner [37]. Both will form vacuoles with laminin positive plasma membranes, but the calcium-mediated fusion may leave a vacuole that may not necessarily contain anything apart from unprocessed cellular debris, which EM-images in this study has demonstrated. To our knowledge, the present study is the first to unambiguously link invaginations to vacuoles with autophagy-related content such as lysosomes and autophagosomes. The dynamics of the invaginations is at present unknown, as they may proceed through the myofiber, creating a vacuole through membrane fusion and then continue, or less likely originate from different points along the sarcolemma and incidentally join at the vacuole. A potentially important effect of the invaginations on autophagy is that the vacuoles at some stage may be exposed to a much higher extra-cellular calcium concentration, which would impede lysosome fusion to autophagosomes as well as well as the autophagic flux possibly through calcium activation of calpain, leading to cleavage of atg5 and inhibition of the atg5-atg12 mediated autophagy induction [38–40].

The systemic pattern of decrease of expression of autophagy-related genes suggested affection of a master regulator of autophagy gene expression. TFEB regulates expression of many autophagy-related genes and is controlled mainly by nutritional state in the form of phosphorylation of phosphoserine 142 and 211 by mTOR [41]. When phosphorylated, TFEB resides in the cytosol, and is unable to translocate to the nucleus. In the event calcium concentration increases, both phosphoserines are dephosphorylated by calcineurin and TFEB is able to translocate to the nucleus and act as a transcription factor [25]. Interestingly, the explanation for the overall decrease of autophagy gene expression could not be found in the normal mechanism for activation/deactivation of TFEB. Evidently, there is a conflict between an increase in calcium concentration and the normal nutritional status in the hypoPP patients. A different issue is the twofold lower expression of TFEB in the hypoPP patients, which could potentially affect all gene targets of TFEB activation and explain the overall decrease in expression of autophagy genes seen in this study. For Pompe disease, activation of TFEB has been suggested as a therapeutic target in order to increase the availability of autophagy-related proteins, and thus increase processing of intracellular waste and lysosomal content [42].

Studies have demonstrated that a master repressor of autophagy also exists, zink-binding protein with KRAB and SCAN domain 3 (ZKSCAN3), controlling the expression of more than 60 genes involved in autophagy and lysosomal biogenesis [43]. However, a later study in ZKSCAN3 knock-out mice found that the expression of many of the same genes as found under ZKSCAN3 repression in Chaucan et als study was unaffected [44]. This obviously present a conflict between the former in vitro and the latter in vivo studies. We have not investigated any role of ZKSCAN3 as it was outside the scope of this study and requires a very comprehensive study to understand how any repression takes place and is regulated.

The present study cannot establish whether the vacuoles presenting disturbed autophagy is related to the decreased gene expression. Clearly, there are many complicating and conflicting factors at play in the muscle of the hypoPP patients. Apart from issues with autophagic flux, autophagic induction may also be affected possibly in more than one way. This study has demonstrated that part of autophagic induction appears to be LC3-independent, and that the concomitant drop in mRNA and protein expression cannot be explained by the increased calcium concentration nor the normal nutritional status/absence of glucose starvation as the activation of the master regulator TFEB was unchanged compared to normal subjects. It is possible that ZKSCAN3 plays a secondary if not a primary role in the decrease/repression of autophagic gene expression, especially as the repression of expression affects not only proteins involved in autophagosome formation and processing, but also endosomes and lysosomes. Systemic decrease in autophagic gene expression has mostly been demonstrated in some cancers, such as melanoma and breast cancer [45, 46] even though autophagy may act both pro- and anti-tumorigenic. Posttranslational control may also play a role in autophagic induction as excellently reviewed by Botti-Millet et al. [47]. As hypoPP patients are not in any nutritional distress, it would be surprising if autophagy was initiated by ULK1/2 activation by the nutritional sensor AMPK due to glucose starvation when the periodic paralytic attacks can be triggered by a carbohydrate-rich meal. Similar, it would not be expected that ULK1/2 was deactivated by mTORC1, which is part of the Akt signalling cascade and involved in activating p70S6K for protein synthesis. However, future studies may look at these initiating factors as well atg1 and atg29, which are known to be involved in induction of autophagy [48, 49] and PKC which activates TFEB and JNK and p38 which inactivates ZKSCAN3 [50].

## Conclusions

In conclusion, we have demonstrated that in hypoPP patients regardless of phenotype, autophagy is negatively affected at two levels: the intermittent arrest of the autophagic process at the early autophagosome/endosome stage and the downregulation of autophagy gene expression. Our results suggest an interdependency between the two and additionally that the failed increase in calcium-driven autophagy gene expression could be due to conflicting signalling between a rise in calcium-concentration and normal nutritional (glucose) status, leading to a decrease rather than an increase in autophagy gene expression. The long-term effect of this dysfunctional autophagy may a muscle loss-of-function as seen in patients with permanent weakness.

## Data Availability

The data supporting the findings of this study are available from the corresponding author upon request.

## Abbreviations

Ca_V_1.1: Voltage-gated calcium channel 1.1
HypoPP: Hypokalemic periodic paralysis
LAMP1/2: Lysosome-associated membrane glycoprotein ½
MAC: Membrane attack complex
Na_V_1.4: Voltage-gated sodium channel 1.4
TEM: Transmission electron microscopy
TFEB: Transcription factor EB; XMEA, X-linked myopathy with excessive autophagy
ZKSCAN3: Zink-binding protein with KRAB and SCAN domain 3
